# Incidence, Pattern and Severity of Adverse Events Following Immunization (AEFIs) Associated With Chadox1 nCOV-19 Corona Virus Vaccine (Recombinant) Among the Healthcare Workers of a Tertiary Care Institute of Eastern Uttar Pradesh, India

**DOI:** 10.7759/cureus.21848

**Published:** 2022-02-02

**Authors:** Bhushan Kamble, MD. Abu Bashar, Chandra Pati Mishra

**Affiliations:** 1 Community and Family Medicine, All India Institute of Medical Sciences, Bibinagar, IND; 2 Community and Family Medicine, All India Institute of Medical Sciences, Gorakhpur, IND; 3 Community Medicine, Institute of Medical Science, Banaras Hindu University, Varanasi, IND

**Keywords:** safety, severity, healthcare workers, aefis, chadox1 ncov-19 corona virus vaccine

## Abstract

Background

In January 2020, the Government of India based on the recommendation of the Drugs Controller General of India (DCGI) and National Technical Advisory Group on Immunization (NTAGI) started the rollout of the COVID-19 vaccine in the country. Two vaccines, ChAdOx1 nCoV-19 coronavirus vaccine (recombinant), i.e., COVISHIELD produced by Serum Institute of India and COVAXIN developed indigenously by Bharat Biotech, were given emergency use authorisation (EUA) by the DCGI.

Methods

In this cohort study, we assessed the incidence, pattern and severity of adverse events following immunization (AEFI) observed among the healthcare workers of a large tertiary care institute in eastern U.P., India vaccinated with ChAdOx1 nCoV-19 Coronavirus vaccine (recombinant) within 30 minutes of vaccination by direct observation.

Results

Out of the total 836 healthcare workers who were vaccinated with the first dose of the vaccine, around 10% experienced any AEFI within the directly observed period. The most common AEFI was pain/tenderness at the injection site experienced by 59.3% of those who experienced any AEFI followed by headache/dizziness (35.3%), itching/rashes at the injection site (8.1%), nausea/vomiting (5.8%) and fever/chills (4.7%). The majority (95.3%) of the AEFIs observed were of minor severity with no serious AEFIs observed as per the WHO severity classification.

Conclusion

ChAdOx1 nCoV-19 Coronavirus vaccine (recombinant) is proven to be safe based on our findings as the majority of AEFIs observed were of minor grade only. However, the vaccine beneficiaries should be strictly observed for a minimum of 30 minutes at the vaccination site to look for any serious AEFI with arrangements to manage the same.

## Introduction

As of June 30th, 2021, coronavirus disease 2019 (COVID-19) has been reported in more than 180 million individuals, and there have been more than 3 million deaths worldwide [1]. Various strategies such as tracking, tracing, testing, quarantine, and lockdown were implemented to combat the pandemic in many countries [2]. Currently, the strategy of mass vaccination against COVID-19 is being implemented in most of the countries across the globe to overcome this global catastrophe. In the absence of any definitive anti-SARS-CoV-2 therapy, mass vaccination against the viral disease may be the only mean for containing this ongoing pandemic.

In India, the COVID-19 vaccination drive was initiated on January 16th, 2021 after approval of two COVID-19 vaccines by the Drugs Controller General of India (DCGI) for restricted emergency use in the country [3]. The priority populations to be vaccinated in the first phase of this drive were the healthcare workers (HCWs) and the frontline workers (FLWs). In the first quarter of the vaccination, HCWs were administered the ChAdOx1 nCoV-19 vaccine/COVISHIELD (AstraZeneca/Serum Institute of India) or BBV152 vaccine/COVAXIN (Bharat Biotech). COVISHIELD is based on a replication-deficient simian adenoviral vector coding the whole length spike glycoprotein (S) of SARS-CoV-2 while COVAXIN is based on the inactivated SARS-CoV-2 platform [4]. Randomized controlled trials (RCTs) have reported an acceptable safety profile for both the vaccines [5, 6]. It is very important to improve the vaccination coverage of the COVID-19 vaccines to achieve the national goal of herd immunity. However, several adverse events associated with COVID-19 vaccines have been reported, including anaphylaxis, transverse myelitis, and deep vein thrombosis [6-8]. Misinformation through the mass media has been the basis of substantial anxiety among people about the safety of the vaccine since the initiation of the vaccination drive [9]. Moreover, there have been very few large-scale research studies on monitoring the adverse events following immunization (AEFI) associated with the COVID-19 vaccines in the Indian population.

We aimed to evaluate the incidence, pattern and severity of AEFIs associated with the ChAdOx1 nCoV-19 vaccine (AstraZeneca/Serum Institute of India) among the HCWs vaccinated at a single center situated in a tertiary care institute of North India during the first phase of vaccination so as to provide a basis to ensure safety of this vaccine during the future national vaccination against COVID-19.

## Materials and methods

Study design and population

A prospective, single-center cohort study using complete enumeration method was conducted from February 1st, 2021 to April 30th, 2021 at the COVID-19 Vaccination Centre, SSL Hospital, Banaras Hindu University, Varanasi, U.P., North India. The study subjects were all the HCWs who were taking the first dose of the ChAdOx1 nCov-19/COVISHIELD vaccine during the time period. As per the government policy, adult HCWs of any age were eligible for the COVID-19 vaccination. Among those who received the vaccination, HCWs who did not give consent for participation in the study were excluded.

Vaccination & AEFI reporting

Information about the ChAdOx1 nCov-19 vaccine and vaccination was notified to all the HCWs of the institute through the head of respective departments by asking the list of HCWs from them for uploading on the COWIN website. Written informed consent was obtained from the HCWs priorly while screening for the vaccination at the vaccination centre before the actual vaccination. For those who consented to undergo vaccination, the vaccination schedules were set from 10 AM to 4 PM daily except Sundays. The vaccination was conducted by dividing the space of the vaccination centre into three sections so that preliminary screening and registration, vaccinations, and AEFI monitoring could be performed simultaneously. The HCWs were asked to fill a preliminary form before vaccination that captured their previous history of vaccination, history of COVID-19 infection, and any known allergies. The vaccine was administered in the deltoid region by the well-trained auxiliary nurse midwife (ANMs)/nurses, and adverse events were directly observed and monitored for 30 minutes at the vaccination centre. Definition of adverse event was considered as defined by the World Health Organization (WHO), i.e., any untoward medical occurrence which follows immunization, and which does not necessarily have a causal relationship with the usage of the vaccine. The event may be any unfavorable or unintended sign, abnormal laboratory finding, symptom or disease [10]. Every HCW was directly observed for at least 30 minutes by one of the investigators who are specialized in identifying the adverse events and those developing any severe or serious AEFI were kept till they were stabilized. After discharge from the vaccination centre, vaccinated HCWs were advised to report any further AEFI in the WhatsApp groups created by the investigators for monitoring the AEFIs on day-to-day basis. If any of the HCW reported any AEFI in the group, he/she was given appropriate advice by the investigators. In case of any emergency arising subsequent to vaccination after the period of direct observation, they were advised to directly call one of the investigators whose number was displayed at the vaccination centre who counselled them regarding the AEFI including prescribing Paracetamol and Ibuprofen for fever and/or body aches. The HCWs were also advised appropriately regarding whether to visit the outpatient clinic of the department of internal medicine or the emergency room (ER) if the adverse events persisted.

Adverse events reporting system

The study questionnaire, developed by the investigators, captured the basic socio-demographic characteristics, history of COVID-19 infection in past, drug and medical history and 10 common adverse events following vaccination. If the HCW did not report any severe/serious AEFI within the direct observation period, he was discharged from the vaccination centre. The questionnaire was pilot tested on 20 HCWs, data of whom were excluded from the actual study, to assess its validity and reliability and suitable changes were made in it based on findings of the pilot study before administering it to the actual study participants.

The AEFI surveillance survey comprised questions about 10 common adverse events and a provision of free-text reporting for any other adverse events not listed in the questionnaire. Solicited local AEFIs included tenderness, pain at rest, redness, and swelling at the injection site. Solicited systemic AEFIs included fatigue, headache, malaise, arthralgia, chills, fever and nausea or vomiting. The severity of the local and systemic AEFIs was graded as per WHO guidelines for classifying AEFIs based on severity [11]. If any of the HCWs visited the outpatient clinics or ERs due to AEFIs, the adverse event was reported to the national COVID-19 vaccination management system on the COWIN portal according to the government's policy.

Statistical analysis

The data were entered into excel sheets and analyzed using SPSS software version 25.0 (IBM Corp., Armonk, NY, USA). The categorical variables were summarized using absolute frequencies and proportions and the quantitative variables were summarized by their means with the standard deviations.

Ethics statement

This study was approved by the Institutional Ethical Committee (IEC) of the Institute of Medical Sciences, BHU, Varanasi (Approval letter No. Dean/2021/EC/2524 dated 12-02-21). All participants provided their written consent prior to taking part in the study. Participation was completely voluntary, and participants were able to withdraw from the study anytime without any consequence. Confidentiality of data was maintained.

## Results

A total of 836 HCWs, out of the 840 HCWs who received the first dose of COVISHIELD vaccine during the study period, gave consent for participation in the study and were monitored for AEFIs at the vaccination center of SS Lal Hospital, BHU, Varanasi, UP. Their background characteristics are mentioned in Table 1.

**Table 1:** Background characteristics of the healthcare workers receiving COVISHIELD vaccine (N=836) *Indian National Rupees

Characteristics	Categories	Frequency	Percentage
Mean age in years ±SD		35.75 ± 9.52	
Gender	Male	573	61.2
	Female	263	38.8
Religion	Hindu	783	93.7
	Muslim	16	1.9
	Sikh	02	0.2
	Christian	35	4.2
Marital status	Unmarried	197	23.6
	Married	636	76.1
	Widowed/Divorced/Separated	03	0.4
Education	Up to matriculation	38	4.5
	Intermediate or Diploma	189	22.6
	Graduation	356	42.6
	Post-graduation & above	243	29.1
Occupation	Doctor	230	27.5
	Nursing Officer/Technician	404	48.3
	OPD/OT/Ward Attendant	202	24.2
Monthly income (in INR*)	Up to 20,000	114	13.6
	20,001-50,000	141	16.9
	50,001-1,00,000	441	52.8
	>1,00,000	140	16.7
Dietary habit	Vegetarian	348	41.6
	Non-vegetarian/Mixed	488	58.4
History of any chronic illness	Present	160	19.1
	Absent	676	81.9
Received any vaccine in the past 4 weeks	Yes	02	0.2
	No	834	99.8
Tested positive for COVID-19 in past	Yes	125	14.9
	No	711	85.1
History of alcohol consumption in the past 12 months	Present	94	11.2
	Absent	742	88.8
History of alcohol consumption within 24 hours	Present	15	1.8
	Absent	821	98.2
History of Smoking/Smokeless tobacco products	Present	102	12.2
	Absent	734	87.8

**Figure 1:**
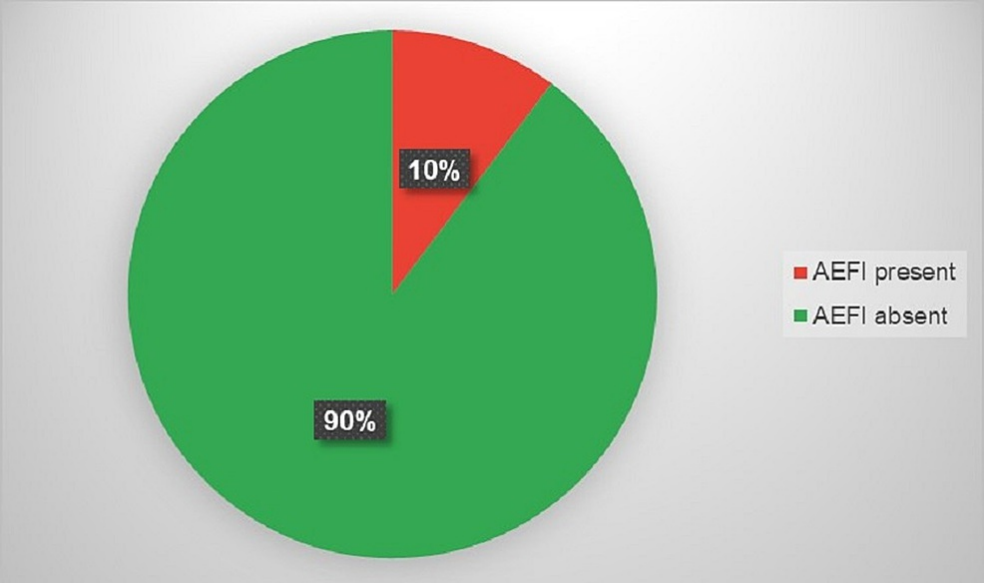
Incidence of AEFI within 30 minutes of vaccination among the healthcare workers (N=836). AEFI: Adverse events following immunization

A total of 86 (10.3%) out of the 836 HCWs reported to experience one or more AEFIs within 30 minutes of vaccination with COVISHIELD vaccine (Figure 1). Out of the 836 HCWs vaccinated with the first dose of COVISHIELD, one (0.12%) developed anaphylaxis.

The most common AEFI was pain/tenderness at the injection site experienced by about two-thirds (59.3%) of the HCWs who reported to have any AEFI followed by headache/dizziness in one third (34.9%), itching/rashes at the injection site (8.1%), nausea/vomiting (5.8%) and fever/chills (4.7%) and the least common AEFIs were increased lacrimation (1.1%) and altered sensorium (1.1%) (Table 2).

**Table 2:** Pattern of AEFIs observed among those healthcare workers who experienced any adverse event within 30 minutes of vaccination with COVISHIELD (n=86) *Multiple responses possible; AEFI: Adverse events following immunization

AEFI observed*	No. (%)
Pain/tenderness at the injection site	51 (59.3)
Headache/Dizziness	30 (34.9)
Itching/rashes at the injection site	7 (8.1)
Nausea/Vomiting	5 (5.8)
Fever/Chills	4 (4.7)
Burning sensation at the injection site	4 (4.7)
Fatigue/weakness	3 (3.5)
Tingling sensation in fingers	2 (2.3)
Increased Lacrimation	1 (1.1)
Altered sensorium	1 (1.1)

**Figure 2:**
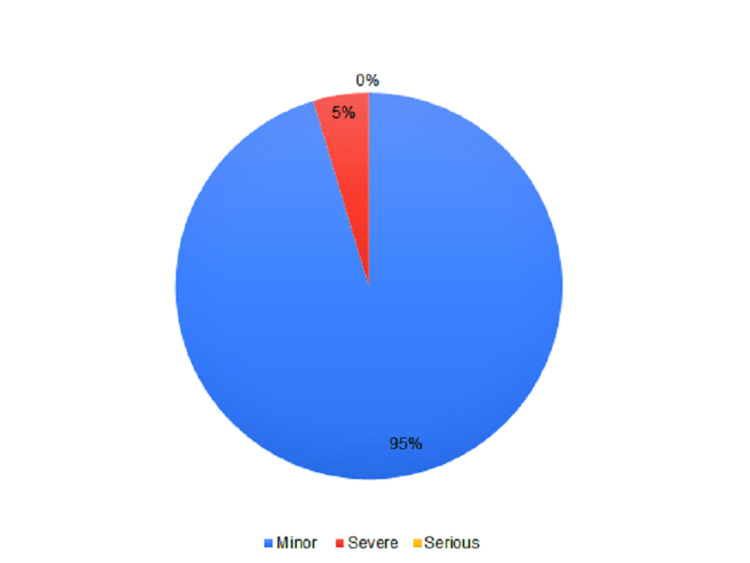
Severity of AEFIs experienced by the healthcare workers within 30 minutes of receiving COVISHIELD vaccine (n=86) AEFI: Adverse events following immunization

Out of the 86 HCWs experiencing any AEFI, 82 (95.3%) had minor grade AEFIs, 4 (4.7%) had severe AEFIs and none had any serious AEFI as per WHO’s severity classification for AEFIs (Figure 2).

## Discussion

Since the beginning of the development of vaccines against COVID-19, concerns have been raised and apprehensions were observed in the populations and sub-groups globally over the adverse events and risks associated with these vaccines. Some adverse events may not have been reported in the clinical trials due to their lower frequency, smaller number of people participating in the trials and other restrictions in the trials. Thus, post-vaccination monitoring of the adverse reactions is important to inform the public and policymakers of the safety and possible severe reactions of the vaccine.

In the present study, we have presented the incidence, pattern and severity of AEFIs within 30 minutes of vaccination observed among the healthcare workers (HCWs) of a tertiary care institute of Northern India vaccinated with ChAdOx1 nCoV-19/COVISHIELD vaccine. As per WHO’s safety surveillance module manual for COVID-19 vaccines, in the context of COVID-19 vaccination, surveillance systems need to be prepared for identifying and responding to both adverse events following immunization (AEFIs) and adverse events of special interest (AESIs) as well as other safety events that may cause public concern, including incidents of substandard or counterfeit vaccines [12].

The incidence of AEFI was found to be 10.3% within the directly observed period among the studied population. We found that the most commonly reported AEFIs after the first dose of the ChAdOx1 nCoV-19/COVISHIELD vaccine were pain/tenderness at the injection site, headache and itching/rashes at the injection site. As per severity, most of the AEFIs were of minor grade (95.3%), only four healthcare workers experienced a severe AEFI. There were no serious events observed requiring hospitalization, and most AEFIs improved before discharging from the vaccination centre.

In an interim analysis of four clinical trials on the ChAdOx1 nCoV-19 vaccine, the most frequently reported adverse reactions were tenderness at the injection site (63.7%), pain at the injection site (54.2%), headache (52.6%), and fatigue (53.1%). The majority of the adverse reactions were mild-to-moderate in severity and usually resolved within a few days of vaccination [6, 13]. Compared to this report, a lower incidence and severity of local and systemic AEFIs were observed in our study.

In a study by Joshi et al. [14] conducted among 1634 Armed Forces Medical Services healthcare workers (HCWs) deployed in Northern India, who took the first dose of ChAdOx1 nCoV-19 Coronavirus vaccine (Recombinant) voluntarily in January-February 2021, 105 vaccine recipients reported at least one AEFI symptoms following COVID-19 vaccination (incidence proportion 6.4%, 95% CI: 5.3%, 7.7%). All AEFIs reported were of minor grade which were managed by tablet paracetamol and subsided after 1-2 days with no severe or serious AEFI being reported among the vaccine recipients.

In a study by Menni et al. [15] from King’s College, London, U.K., systemic side-effects were reported by 33.7% of the participants and local side-effects were reported by 58.7% of the participants after the first dose of ChAdOx1 nCoV-19, both figures much higher than that observed in our study.

Huh et al. [16] reported that the incidence of anaphylaxis associated with vaccination tended to increase with time in Korea. As of March 26, 2021, according to the reports of adverse reactions after vaccination against COVID-19 in Korea, 96 suspected cases of anaphylaxis were reported among 771,284 individuals (0.01%) receiving the first dose of the vaccine [17]. However, in our study, only one HCW presented with an acute allergic reaction with breathlessness and hypotension, which was resolved spontaneously. Moreover, no serious AEFIs requiring hospitalization or death were reported. These results also are consistent with the results of the ChAdOx1 nCoV-19 vaccination among HCWs in Nepal, Afghanistan and South Korea [18-20]. Such mild-to-moderate AEFIs are acceptable during immunization against COVID-19. Results of our study would be helpful in addressing the vaccine hesitancy caused by the concerns about severe adverse events associated with the COVID-19 vaccine.

Strengths and limitations

The main strength of the study was the very little chances of non-response bias as all the healthcare workers who received the COISHIELD vaccine were directly observed. However, the current study only reports after administration of the first dose of the vaccine, which limits the information about the incidence, pattern and severity of side effects reported after the second dose of vaccine and the information after the second dose of the vaccine is yet to be explored in the population under study. Being a single-center study, it also makes it difficult to generalize the findings; however, the findings of this study could provide a useful insight into the situation and may play an important role in reducing vaccine hesitancy among the public.

## Conclusions

In conclusion, only 10.3% of the health care workers (HCWs) from a tertiary care institute of U.P., North India receiving the first dose of ChAdOx1 nCoV-19/COVISHIELD experienced any AEFI. Mild-to-moderate pain or tenderness at the injection site and headache or dizziness were the most frequently reported AEFIs. Among the healthcare workers who experienced any AEFI, the majority (95.3%) had minor AEFIs and there were no serious AEFIs that required hospitalization or resulted in death. To develop a novel vaccination strategy against COVID-19 and to improve its coverage, the sharing of accurate and abundant information regarding vaccine safety through post-vaccination surveillance of AEFIs is of utmost importance.
